# Simulation of malaria epidemiology and control in the highlands of western Kenya

**DOI:** 10.1186/1475-2875-11-357

**Published:** 2012-10-29

**Authors:** Erin M Stuckey, Jennifer C Stevenson, Mary K Cooke, Chrispin Owaga, Elizabeth Marube, George Oando, Diggory Hardy, Chris Drakeley, Thomas A Smith, Jonathan Cox, Nakul Chitnis

**Affiliations:** 1 Department of Epidemiology and Public Health, Swiss Tropical and Public Health Institute, Socinstrasse 57, Postfach, Basel, CH-4002, Switzerland; 2 University of Basel, Basel, Switzerland; 3 Faculty of Infectious & Tropical Diseases, London School of Hygiene and Tropical Medicine, Keppel St WC1E 7HT, London, UK; 4 Centre for Global Health Research, Kenya Medical Research Institute/Centers for Disease Control and Prevention, Kisumu, Kenya

**Keywords:** Simulation, Kenya, EIR, Mathematical Modelling, Sensitivity analysis, Malaria, OpenMalaria

## Abstract

**Background:**

Models of *Plasmodium falciparum* malaria epidemiology that provide realistic quantitative predictions of likely epidemiological outcomes of existing vector control strategies have the potential to assist in planning for the control and elimination of malaria. This work investigates the applicability of mathematical modelling of malaria transmission dynamics in Rachuonyo South, a district with low, unstable transmission in the highlands of western Kenya.

**Methods:**

Individual-based stochastic simulation models of malaria in humans and a deterministic model of malaria in mosquitoes as part of the OpenMalaria platform were parameterized to create a scenario for the study area based on data from ongoing field studies and available literature. The scenario was simulated for a period of two years with a population of 10,000 individuals and validated against malaria survey data from Rachuonyo South. Simulations were repeated with multiple random seeds and an ensemble of 14 model variants to address stochasticity and model uncertainty. A one-dimensional sensitivity analysis was conducted to address parameter uncertainty.

**Results:**

The scenario was able to reproduce the seasonal pattern of the entomological inoculation rate (EIR) and patent infections observed in an all-age cohort of individuals sampled monthly for one year. Using an EIR estimated from serology to parameterize the scenario resulted in a closer fit to parasite prevalence than an EIR estimated using entomological methods. The scenario parameterization was most sensitive to changes in the timing and effectiveness of indoor residual spraying (IRS) and the method used to detect *P. falciparum* in humans. It was less sensitive than expected to changes in vector biting behaviour and climatic patterns.

**Conclusions:**

The OpenMalaria model of *P. falciparum* transmission can be used to simulate the impact of different combinations of current and potential control interventions to help plan malaria control in this low transmission setting. In this setting and for these scenarios, results were highly sensitive to transmission, vector exophagy, exophily and susceptibility to IRS, and the detection method used for surveillance. The level of accuracy of the results will thus depend upon the precision of estimates for each. New methods for analysing and evaluating uncertainty in simulation results will enhance the usefulness of simulations for malaria control decision-making. Improved measurement tools and increased primary data collection will enhance model parameterization and epidemiological monitoring. Further research is needed on the relationship between malaria indices to identify the best way to quantify transmission in low transmission settings. Measuring EIR through mosquito collection may not be the optimal way to estimate transmission intensity in areas with low, unstable transmission.

## Background

### Rationale for work

In order to make informed decisions for malaria control, programme managers require information on the optimal mix of intervention strategies tailored to specific transmission patterns of malaria [1-3]. This information is often unavailable due to the difficulty in measuring rates of malaria transmission and determining the impact of control interventions on transmission. While the efficacy of individual malaria control interventions in reducing morbidity and mortality in western Kenya has been demonstrated by field trials [4,5], there have been fewer studies investigating the effects across a range of transmission intensities or for combinations of interventions [6,7].

Since 2008, a number of epidemiological and entomological studies have been carried out in Rachuonyo South, Kenya, as part of the Malaria Transmission Consortium (MTC). The availability of data from these and other studies presents an opportunity for site-specific parameterization of models of malaria transmission. The results of these model simulations can be translated into evidence-based decision making for malaria control programme managers. This project applies individual-based stochastic models of malaria to MTC sites with transmission data to simulate the impact of a range of malaria control strategies.

### Study area

**Figure 1:**
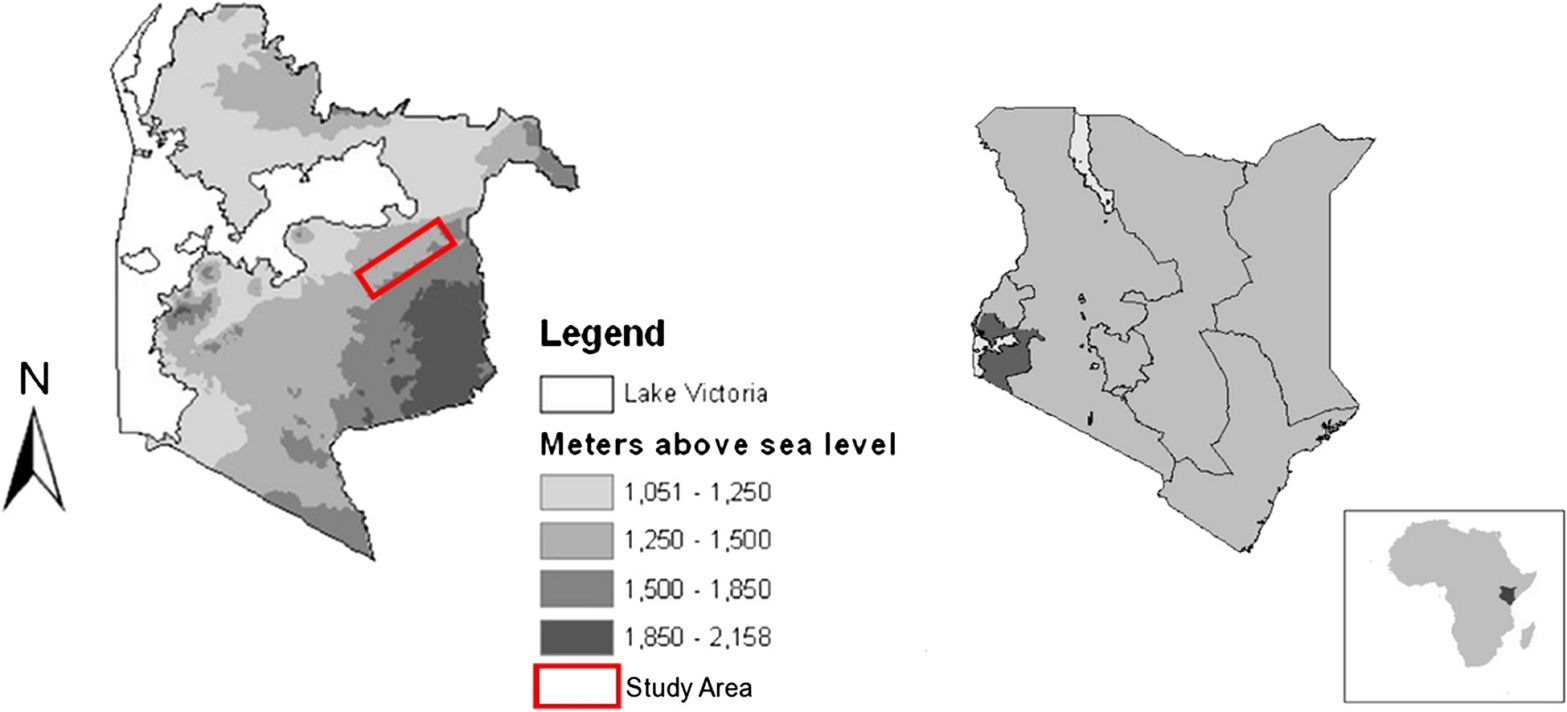
**Map of the study area.** Map of **a**) Location and elevation of the study area in Rachuonyo South district; and **b**) Location of Nyanza Province in relation to Kenya.

**Figure 2:**
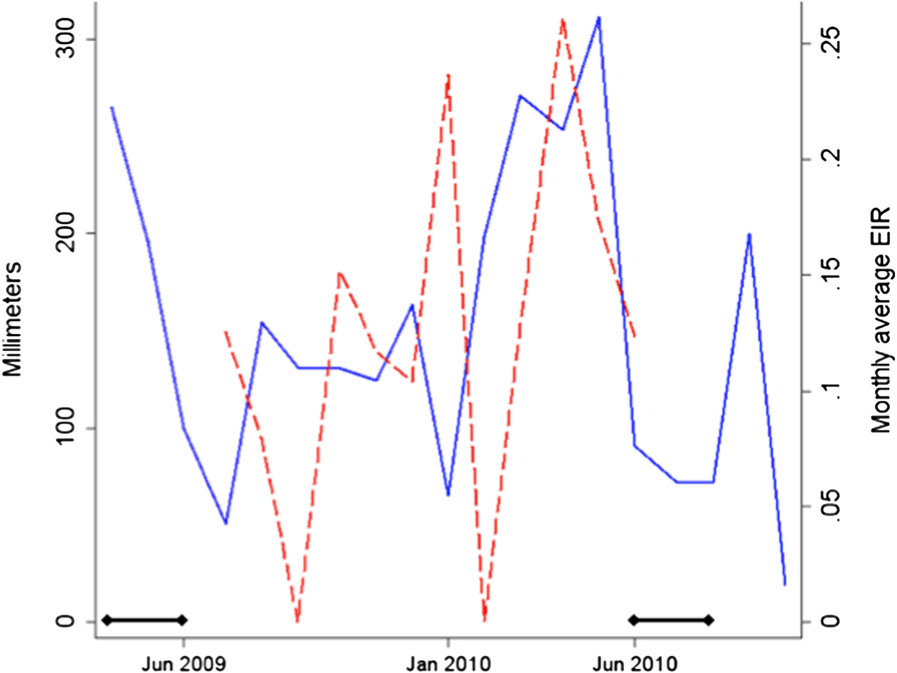
**Seasonal patterns of rainfall, estimated EIR, and timing of IRS interventions.** The rainfall pattern (solid blue line) collected by the weather station at Kogalo Primary School, Kowuor Location, Rachuonyo South that informed the estimated seasonal pattern of the EIR (dashed red line) in Rachuonyo South district over the period June 2009 – June 2010. The black-capped bars indicate the timing of the 2009 – 2010 deployment of IRS in Rachuonyo South district.

Rachuonyo South district is situated in Nyanza province, bordering Lake Victoria in western Kenya (Figure 1) and encompasses an area of 930km^2^. The main MTC study site is located in the south west of the district and represents a highland “fringe” area (1,400-1,600 meters above sea level). Ethnicity in Rachuonyo South is predominantly the Luo ethnic group. Residents depend upon farming and cattle and goat herding for subsistence. Homesteads are distributed broadly across a rolling landscape intersected with small streams and rivers. Total annual rainfall in this area averages 1,200 mm per year (Figure 2) while average daily temperatures range from 17-27°C. The area is characterized by generally low malaria endemicity with marked seasonal and inter-annual variations in transmission [8,9]. 

The main malaria vectors in the highlands were previously recorded to be *Anopheles gambiae sensu stricto*, *Anopheles arabiensis* and *Anopheles funestus*[10,11]. In recent years, there is evidence that *An. gambiae s.s.* is disappearing from lowlands Nyanza leaving *An. arabiensis* as the predominant species within the *An. gambiae sensu lato* complex [12] and *An. funestus* as the primary *Plasmodium falciparum* vector (Stevenson*, personal communication)*. These changes are most likely due to intensive targeting of malaria control interventions, but climatic factors may also have played a role [12-14].

In western Kenya indoor residual spraying (IRS) campaigns were carried out in the Kericho district in the 1940s and Nandi district in the 1950s (using dichlorodiphenyltrichloroethane (DDT) and dieldrin, respectively). It is thought that malaria transmission was largely eliminated from large portions of the highlands as a result [15,16]. While epidemics re-emerged in the 1980s [17,18], it was not until after the year 2000 that routine, large-scale vector control interventions were introduced in these areas.

The main control methods used today in the epidemic highland areas include mass-distribution of long-lasting insecticide-treated nets (LLINs), IRS with pyrethroids, and prompt and effective treatment of malaria [19-21]. Artemisinin-based combination therapy (ACT), specifically artemether-lumefantrine (AL) was adopted as the first line treatment drug in 2006 following a decline in efficacy of sulphadoxine-pyrimethamine (SP) and amodiaquine, the previous first and second line treatments, respectively [19]. In 2006 and 2011 Rachuonyo South was included within the Kenyan national mass distribution LLIN campaign and distribution continues through antenatal clinics, child welfare clinics, and comprehensive care clinics for people living with HIV. Since 2005 Rachuonyo South has been targeted for universal coverage of IRS once per year in advance of the main transmission season. Different formulations of pyrethroid insecticide have been used over the years with lambdacyhalothrin (ICON) used in 2009, alphacypermethrin (FENDONA) used in 2010, ICON again in 2011, and 2012 started with ICON and then switched to deltamethrin.

## Methods

### OpenMalaria transmission model

A team at the Swiss Tropical and Public Health Institute (Swiss TPH) and Liverpool School of Tropical Medicine (LSTM) has developed stochastic simulation models of transmission of malaria based on the simulation of infection in individuals that are able to simulate the impact (cost-effectiveness, clinical, epidemiological and entomological) of numerous intervention strategies for malaria control [22-26]. These models form part of the OpenMalaria platform that makes the considerable code base written in C++ freely available online [27]. Users are able to carry out predictive simulations either via a downloadable stand-alone programme or via a volunteer grid computing resource and semi-automated experiment design and analysis platform capable of handling entire experiments of 10,000-100,000 scenarios.

Individual infections are simulated by stochastic series of parasite densities, which determine an individual’s morbidity and mortality risks as well as their infectiousness to vectors [22,27]. The simulated infections are nested within simulations of individuals in human populations, and linked to a model of transmission of malaria between humans and mosquitoes and to models of interventions [22,23,27]. The transmission model is based on a periodically-forced difference equation model for malaria mosquitoes feeding on, infecting and getting infected from a heterogeneous population of hosts [26]. These dynamics are calibrated by a seasonal pattern of EIR for each mosquito species assuming that in the absence of interventions EIR seasonality is fixed across years [26]. Simulations are run for one human life span to induce an “equilibrium” level of immunity in the population. Subsequent dynamics are used to predict available malaria outcomes, such as patterns of infection in humans or patterns of disease by age and season, which can then be compared to field data.

The details of the methods to build and parameterize the transmission model used in this project have been published elsewhere [22-26] and therefore are not covered in this paper. In this paper the model components described above are employed to an ensemble of 14 model variants for malaria in humans to address stochasticity and model uncertainty [25]. Simulations were repeated with multiple random seeds to address parameter uncertainty.

### Model parameterization

The models included in the OpenMalaria platform were initially parameterized from published data from Namawala, Tanzania [22-28]; 61 data sets were used to optimize certain parameters [22-26]. To update the parameterization for the Rachuonyo South scenario, data collected as part of the MTC project in the study area was the first choice to use for the model parameters. A description of these studies and how they were used to parameterize the model can be found in Additional file 1.

### MTC field studies

A number of field studies were carried out in Kisii and Rachuonyo South districts between 2009 and 2011 with the goal of establishing an evidence base to help malaria control programme managers monitor malaria transmission and implement and adjust malaria control interventions. Data from these studies are currently being analysed and will be described in detail in forthcoming publications. For the purposes of the modelling work described in this paper, the datasets used are described in Table 1.

**Table 1:** Use of datasets from MTC Field Studies

**Study**	**Timeframe**	**Study population**	**Type and purpose of data used**
Community-based cohort	May 2009 – June 2010	3235 people of all ages above 6 months	Monthly malaria prevalence for model validation Coverage levels of LLINs and IRS for model simulation
Community-based cross sectional	February 2009	2607 individuals	Coverage levels of LLINs and IRS for district-level sensitivity analysis
Community-based cross sectional	July 2009	3587 individuals	
4 x 4 Latin square entomological	2009 - 2010	8 households	Vector species distribution for transmission model
Pyrethrum spray catch entomological	September 2009 - present	200 households	Indoor vs. outdoor vector biting behaviour in areas with or without indoor residual spraying and/or insecticide treated nets
Weather station	Continuous	Kogalo Primary School, Kowuor Location, Rachuonyo South	Seasonality of rainfall and temperature to adjust entomological parameters

Where data were not available from MTC surveys, parameter inputs were identified via a literature review of publications using the PubMed electronic database using the key words “Kenya, Nyanza, Rachuonyo, western Kenya, malaria, *Plasmodium falciparum*, transmission, antimalarials, artemether- lumfantrine, insecticide residual spraying, insecticide-treated nets, larviciding, intermittent preventive treatment, modelling, malaria incidence, treatment seeking, mosquito resting duration, extrinsic incubation period, Anopheles.” An internet review was also conducted on the websites for the Kenyan Ministry of Health, Division of Malaria Control, the National Bureaus of Statistics, and the National Demographic Health Surveys. The sources were prioritized in the following strata in order of precedence: study area districts MTC data collection, study area districts existing literature, study area provincial data, national level data, existing model parameterization. Where more than one data source was found within any one stratum the study with the closest site characteristics or, where applicable, date of data collection closest to that of the MTC studies was used.

To determine the annual average EIR, the transmission parameter in the model, seroconversion rates using the MSP-1 antigen were estimated from the July 2009 cross-sectional survey as described in Drakeley et al. 2005 [29] and derived EIR equivalents were calculated as described in Corran et al. 2007 [30]. The average monthly EIR values used to calibrate the seasonal pattern of transmission in the scenario were calculated by separating the annual average EIR from existing literature for a neighboring district into the monthly proportion of rainfall in Rachuonyo South recorded by the Kogalo weather station so that the peak malaria transmission month corresponded to one month later than the peak rainfall month (Figure 2). Because the annual average EIR is based on serology, the model incorporates the overall temperature and humidity effects but excludes the seasonality of these effects.

In practice, many of the entomological and health system parameters were based on data from elsewhere used in other modelling exercises [26-32] as they are thought to be fairly standard across anopheline species and anti-malarials. However, because several entomological parameters are sensitive to temperature, particularly the extrinsic incubation period (EIP) and mosquito resting duration [33,34], these values were adjusted for each study area based on the average annual temperature collected by the Kogalo weather station. Also, the latest data from the study site challenges the assumption that vectors are normally predominantly endophilic and endophagic [35]. For the purposes of this experiment, emphasis was placed on overall vector biting behaviour rather than simulating individual species. This was due to the design of the entomological field studies for which results were available at the time of model parameterization that focused on indoor/outdoor species composition and trap evaluation rather than the biting behaviour within individual species. The efficacy of LLINs and IRS were adjusted to affect the indoor mosquitoes but not the outdoor mosquitoes and the proportion of bites on a human compared to other mammals was reduced for the outdoor mosquitoes.

The monitoring measures serving as the outputs simulated by the model were chosen based on the indicators of malaria transmission measured by the field studies described above.

### Simulation

Before the main simulation, the scenario was run for one human life span to ensure each simulated individual acquired the expected natural immunity for his or her age. The fitting of the dynamic EIR in the transmission model to the pre-intervention calibration EIR was done during the last five years of the life span simulation. A subpopulation was considered as a cohort and received mass drug administration (MDA) at the beginning of the main simulation, to “mimic” the MTC cohort study conditions, where participants were given a course of the first-line malaria treatment upon enrollment into the study to clear any existing malaria parasites. Finally, the effect of interventions on epidemiological outcomes of malaria in the full population of the study area was simulated for two years.

### Validation and sensitivity analysis

The project addresses uncertainty on three levels: stochasticity, model uncertainty, and parameter uncertainty. Each simulation was repeated by the OpenMalaria simulator on an ensemble of 14 model variants using ten random seeds in order to address model uncertainty and stochasticity. Results in the form of graphs from the ensemble of model variants were visually analysed and compared to observed data from the study areas using Stata (version 11; College Station, TX, USA). Further analysis of the scenario simulation and observed data for the selected impact measures was conducted using Stata. The proportion of simulation results falling within the 95% confidence intervals of the observed cohort data was measured in order to assess goodness of fit.

A sensitivity analysis to address parameter uncertainty was driven by results of the visual comparison of stochasticity. Elements of the model central to the epidemiology and control of malaria in this particular study area were identified based on whether there was uncertainty about parameter estimates and their potential impact on the composition and behaviour of vectors, effectiveness of interventions, and population-level monitoring. These included effectiveness of IRS, indoor versus outdoor biting behaviour of the vectors, the detection limit of the survey method used for malaria in humans, annual average EIR, and climate and weather patterns affecting vector biology and parasite development in the vector. Parameters were altered one at a time and results analysed by comparing the simulated number of cases per person per year for each scenario to those of the baseline parameterization.

## Results

### Model design and baseline scenario parameterization

The following parameter estimates are based on currently unpublished data from the MTC studies described above in Table 1. The key entomological feature of the scenario involves one primary malaria vector that bites and rests outdoors 62% of the time. At the time of enrollment into the cohort, 30.3% of the cohort population slept under a net the previous night and 69.3% of survey households received IRS. The IRS deployment schedule happened yearly over a period of two months as described above in the Background section. The annual mean temperature in Rachuonyo South from 2009 to 2010 was recorded as 20.3 degrees Celsius, setting the estimate of the extrinsic incubation period of *An. gambiae* at 14 days and the resting duration 3 days. Malaria transmission is highly variable following two distinct rainy seasons. The EIR is unstable with a last recorded value from an entomological survey of 0.4 infectious bites per person per year [10]. This study was conducted in neighboring Kisii district before LLIN and IRS scale-up in 2006. More recent results from the July 2009 MTC cross sectional study estimate an EIR of 1.5 infectious bites per person per year based on serological data (Table 2). 

**Table 2:** Malaria transmission parameter values*

**Month**	**Average EIR**	**Month**	**Average EIR**
January	0.003	July	0.079
February	0.129	August	0
March	0.261	September	0.152
April	0.173	October	0.117
May	0.123	November	0.104
June	0.125	December	0.236
Annual average EIR		1.5**	

*All values based on Shililu 1998[36]*, Ndenga 2006*[10]*and data from the Kogalo weather station unless otherwise noted*.

**Annual average EIR based on seroconversion rates as described in Drakeley et al. *2005*[29]*of samples from a cross sectional survey of 3,587 individuals of all ages conducted in the study area in June 2009. EIR equivalents were derived as described in Corran* et al. *2007*[30].

For OpenMalaria to simulate dynamics of the study population, code was included in the scenario to select a cohort representing 15% of the total population over one year old matching the cohort enrollment criteria, all of whom received a course of anti-malarials at the start of the survey period. The validation of the model uses the model outputs from only this cohort, while the remaining simulations represent the larger study area population of 10,000 individuals. The details of the values used to parameterize the model along with their sources can be found in Additional Files 2, 3, 4, 5, 6, 7.

### Simulation and validation

**Figure 3:**
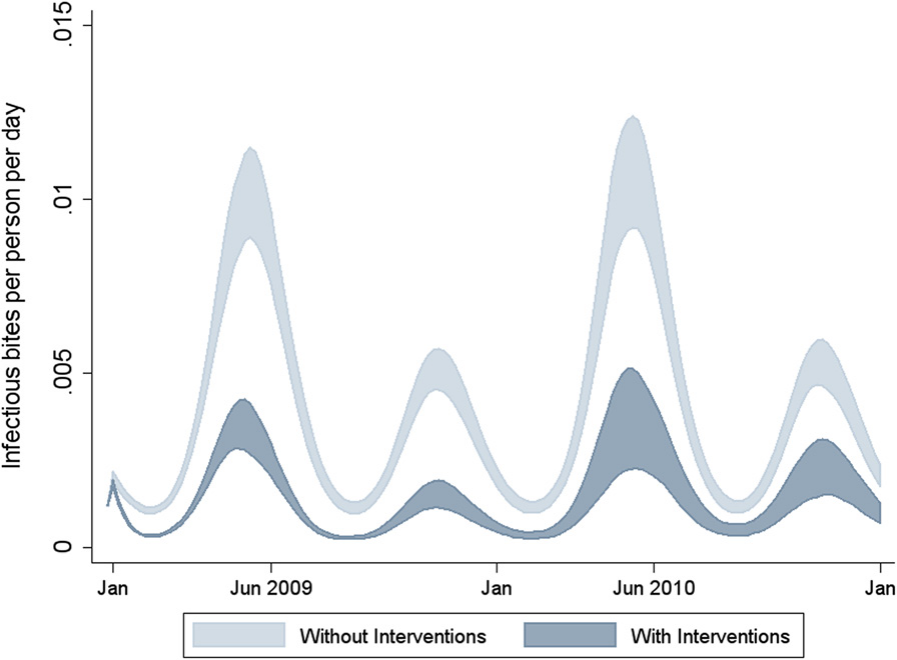
**Simulated seasonal transmission dynamics with and without interventions.** Baseline model simulation of EIR on a population of 10,000 individuals for two years using 10 random seeds for each of the 14 OpenMalaria model variants with (dark blue shaded area) and without (light blue shaded area) interventions in Rachuonyo South district. The daily EIR is calibrated from monthly EIR values that are smoothed out with a Fourier transform to only include an annual and biannual cycle as described in Chitnis et al. 2012 [26]. The shaded areas represent the range of results from the 140 simulations.

**Figure 4:**
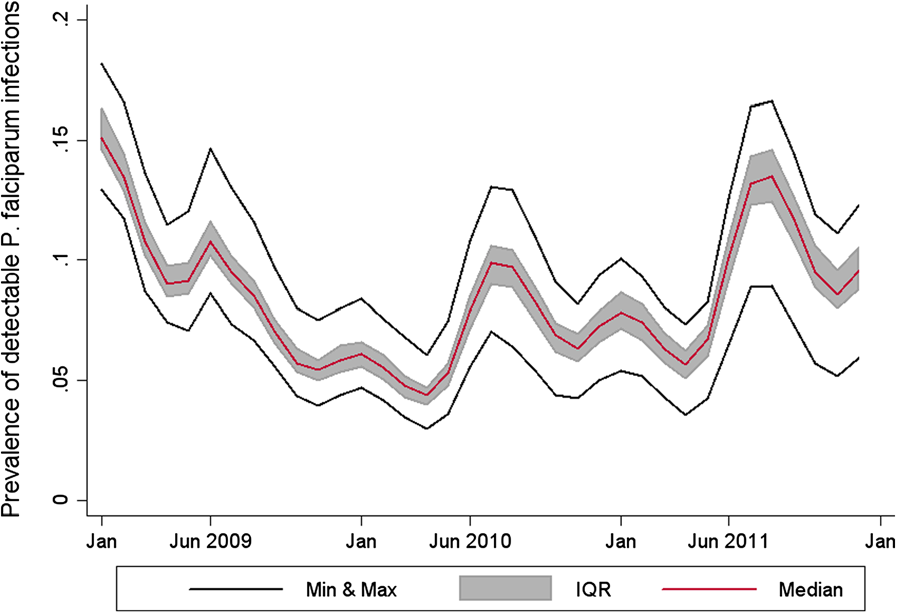
**District-level simulation of number of detectable *P. falciparum *infections. **Simulated number of *P. falciparum *infections as detected by a rapid diagnostic test (RDT) in a population of 10,000 individuals. The simulation ran for two years using 10 random seeds for each of the 14 OpenMalaria model variants. The red line shows the median value of the 140 simulations at each time point. The shaded grey area shows the interquartile range, and the two black lines show the maximum and minimum value at each time point.

OpenMalaria is able to simulate the seasonality and level of the EIR for the Rachuonyo South scenario with greater stochasticity in the peak months and in the scenario with observed interventions (Figure 3). Simulations show prevalence between 5.58% and 10.81% in Rachuonyo South’s peak transmission month and between 2.99% and 6.04% in the lowest transmission month (Figure 4).

**Figure 5:**
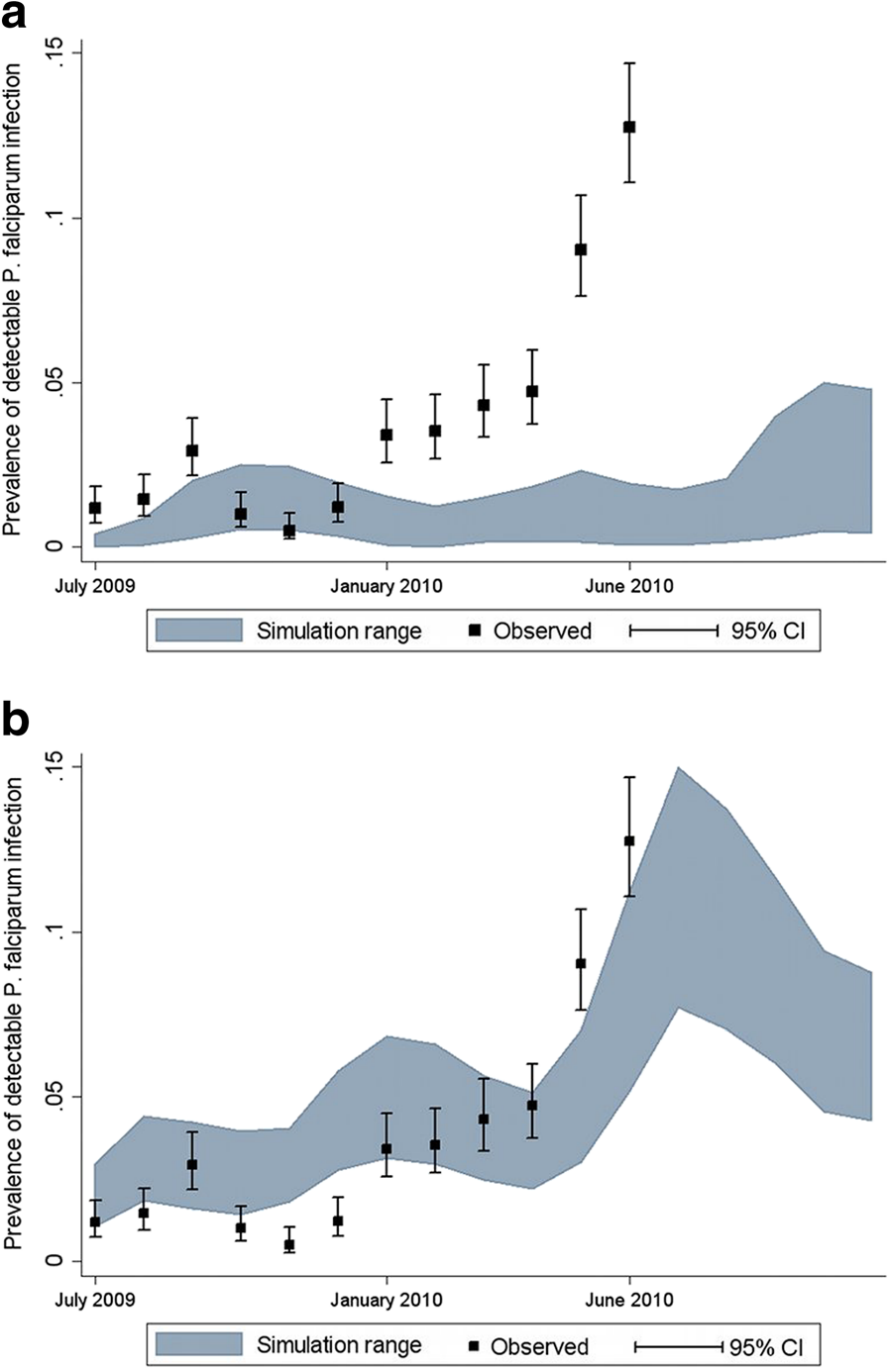
**Model validation with observed cohort prevalence data.** Simulated vs. observed proportion of a cohort of 1,655 individuals in Rachuonyo South District with detectable *P. falciparum* infection for EIR values derived from **a**) entomological studies (0.4 infectious bites per person per year, seasonality from neighboring district; and **b**) serology (1.5 infectious bites per person per year from one primary vector, seasonality from study site weather station). All simulations ran for three years for each of the 14 OpenMalaria model variants. The black squares represent the mean number of patent infections observed in the cohort at each time point. The black-capped bars represent the upper and lower 95% confidence intervals of the observed mean. The shaded area represents the range of results from the 140 simulations. The source of observed data is the MTC cohort study described in the Methods section.

The Figures 5a and b compare the simulation of *P. falciparum* prevalence in the population with observed data from the MTC cohort study conducted from June 2009 – June 2010 in Rachuonyo South District as detected by a rapid diagnostic test (RDT) using EIR values derived from entomological studies (0.4 infectious bites per person per year, seasonality from neighboring district, Table 2) versus serology (1.5 infectious bites per person per year, seasonality from study site weather station). The prevalence was especially high in June of 2010, possibly due to a combination of more rainfall than normal during the rainy season and rollout of IRS at a later month compared to the previous year.

While the model is able to predict the level of prevalence in both scenarios, using an EIR from serology and seasonality from weather station data represents a visually better fit with both level of overall and seasonal prevalence compared to using an EIR and seasonality from entomology. Using the proportion of simulation runs falling within the 95% confidence intervals of the observed cohort data as a benchmark for comparing simulation results, the final scenario was able to improve both the number of months (six months with more than 30% of simulations runs predicted compared to three months, n=12) and the proportion of total simulation runs (29.9% vs. 14.6%, n=1,680).

### Sensitivity analysis

#### Indoor residual spraying

The two main malaria control measures in the study area are distribution of LLINs and IRS. While net use is assumed constant over the time frame of the simulation, IRS is a timed intervention that occurred between April and May of 2009 and June and July of 2010 (Figure 2). To simulate the impact of IRS effectiveness at killing and deterring vectors and the rate at which the insecticide decays on model predictions, scenarios were created to simulate very high and very low IRS effectiveness (Table 3).

**Table 3:** IRS scenario variables

**Variable**	**Description**	**Level**
**IRS description**	IRS decay half-life	**Baseline: 4 months**
		Highly effective: 9 months
		Insecticide resistance: 2 months
	IRS deterrent effect	**Baseline: 0.1116**
		Highly effective: 0.8632125
		Insecticide resistance: 0.1
	IRS postprandial killing effect	**Baseline: 0.2772**
		Highly effective: 0.8
		Insecticide resistance: 0.1

**Figure 6:**
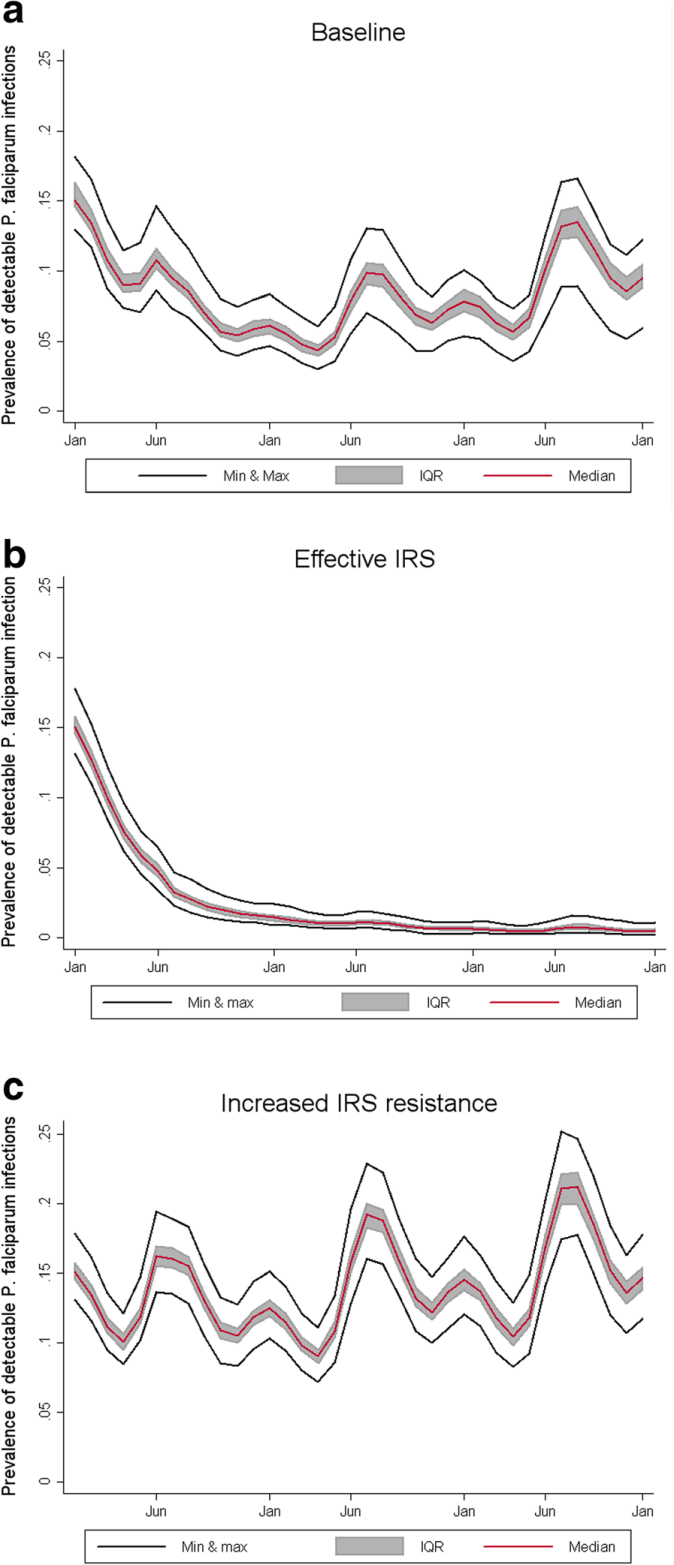
**Sensitivity analysis of IRS effectiveness.** Effect of **b**) highly effective IRS intervention with a half-life decay of 9 months and a killing effect of 80% and **c**) ineffective IRS intervention with a half-life decay of 2 months and a killing effect of 10% on the simulated number of *P. falciparum* infections as detected by RDT in a cohort of 10,000 individuals in Rachuonyo South district compared to **a**) baseline model with half-life decay of 4 months and a killing effect of 27.72%. The simulation ran for two years using 10 random seeds for each of the 14 OpenMalaria model variants. The red line shows the median value of the 140 simulations at each time point. The shaded grey area shows the interquartile range, and the two black lines show the maximum and minimum value at each time point.

Compared to the baseline, increasing the duration and effectiveness of IRS had the effect of greatly reducing the simulated number of patent infections (Figure 6b). While prevalence is greatly reduced, transmission is never completely interrupted even in the scenario simulating highly effective IRS.

#### Biting behaviour

To study the effects of changes in vector diversity and biting behaviour, different scenarios of proportion of indoor vs. outdoor biting are considered. The baseline scenario assumes one primary vector species which bites outdoors 64% of the time and indoors 36% of the time. The experiment includes one scenario with increased exophagy with 74% of transmission occurring outdoors and 26% of transmission occurring indoors and a second scenario with transmission is split equally indoors and outdoors. This is modeled by reducing the effectiveness of vector control interventions.

**Figure 7:**
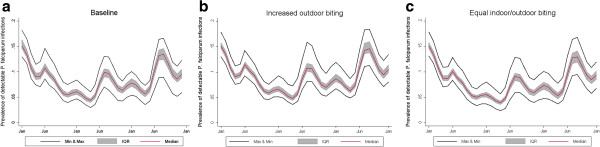
**Sensitivity analysis of biting behavior.** Effect of changing biting behaviour on the simulated number of *P. falciparum* infections as detected by RDT in a population of 10,000 individuals for **a**) baseline model with one primary vector species 64% exophagy and 36% endophagy, **b**) increased exophagy (74%) and **c**) equal exo- and endophagy. The simulation ran for two years using 10 seeds for each of the 14 OpenMalaria model variants. The red line shows the median value of the 140 simulations at each time point. The shaded grey area shows the interquartile range, and the two black lines show the maximum and minimum value at each time point.

The scenario in which the biting behaviour of a single vector species is altered and a greater proportion of EIR is due to indoor biting (increased from 36% to 50%) shows a reduction in prevalence (Figure 7c). This is because the indoor mosquitoes would be affected by the IRS campaigns conducted in April – May of the first year and June – July of the second year. The scenario with a greater proportion of transmission from outdoor biting shows a similar level of transmission during the low season but greater amplitude in peak months (Figure 7b).

#### Survey detection limit

**Figure 8:**
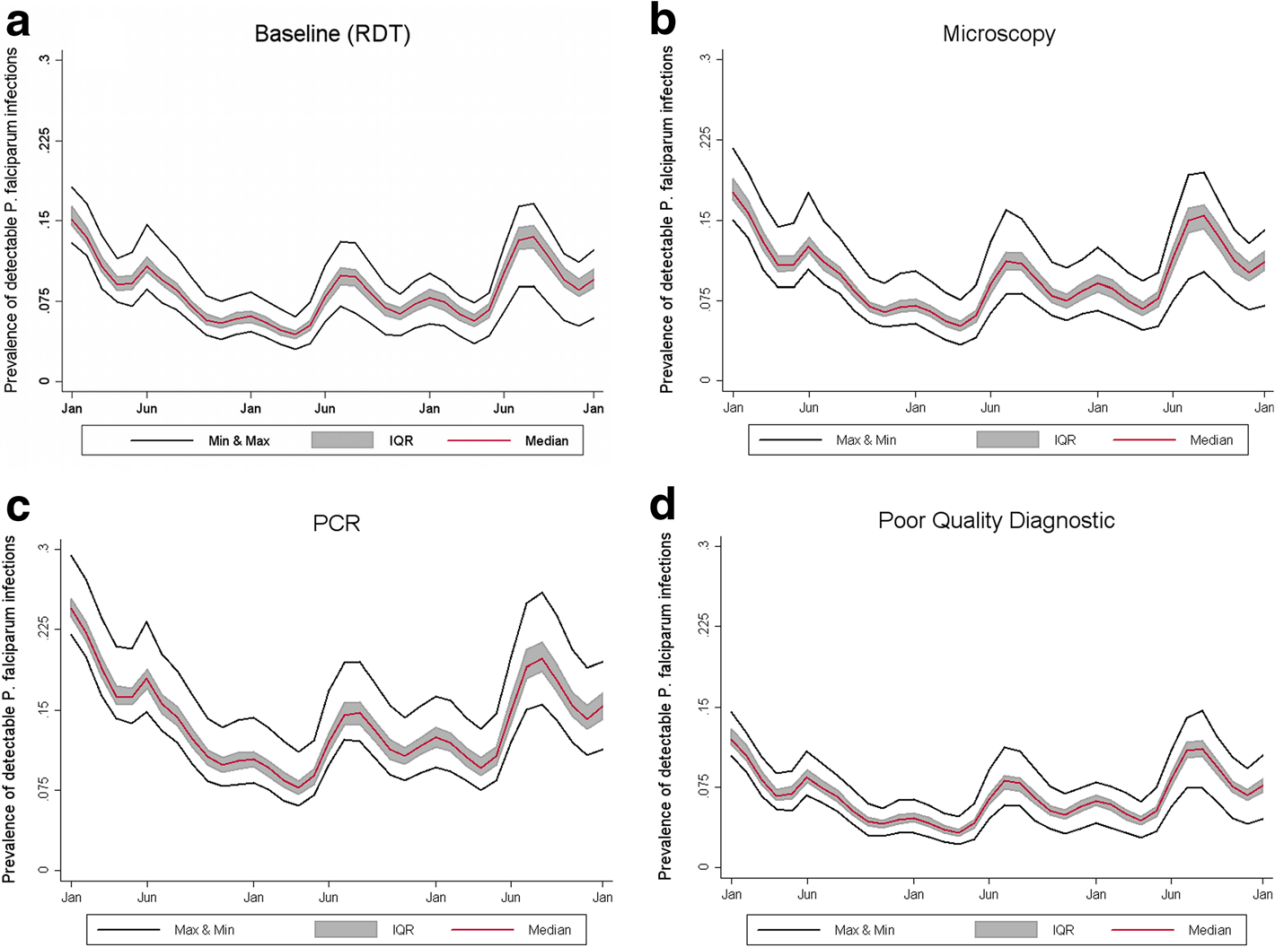
**Sensitivity analysis of detection limit of monitoring methods.** Effect of changing the detection limit (number of parasites per microliter) at which the survey is able to detect *P. falciparum* infection on the simulated number of *P. falciparum* infections in a population of 10,000 individuals for **a**) baseline model with a detection limit of 200, equivalent to RDT; **b**) detection limit of 40, equivalent to PCR; **c**) detection limit of 100, equivalent to skilled microscopy; and d) detection limit of 500, equivalent to a poor quality diagnostic. The simulation ran for two years using 10 seeds for each of the 14 OpenMalaria model variants. The red line shows the median value of the 140 simulations at each time point. The shaded grey area shows the interquartile range, and the two black lines show the maximum and minimum value at each time point.

To address the model’s sensitivity to the ability of a given test to detect a *P. falciparum* infection, an experiment was created to mimic the detection limits of polymerase chain reaction (PCR), skilled microscopy, and a low-quality diagnostic such as a poor-quality RDT or unskilled microscopy (Table 4). The number of simulated infections decreases with higher detection limits, as does the stochasticity of the predictions (Figure 8). This indicates a population that has a considerable proportion of infections occurring characterized by low parasitaemia.

**Table 4:** Detection Limit scenario variables

**Variable**	**Description**	**Level**
**Detection Limit**	Parasites per microliter	PCR: 10
		Skilled microscopy: 100
		**Baseline (RDT): 200**
		Low-quality diagnostic: 500

#### EIR and climatic patterns

In order to account for differences in collection and calculation method as well as micro-variations in EIR within the study area, an experiment was conducted with varying levels of the annual average EIR while keeping the seasonal pattern the same over the baseline. This includes a scenario with a low EIR value that was measured in an neighbouring district a slightly higher altitude before large-scale control programmes were implemented in 2006, two scenarios with medium EIR (one equal to double the recorded value and one equal to the recorded value in the neighboring lowland districts), and a larger EIR. OpenMalaria is able to simulate the scenarios with EIRs of 7 and 20 with less stochasticity than the scenarios with smaller EIRs.

**Figure 9:**
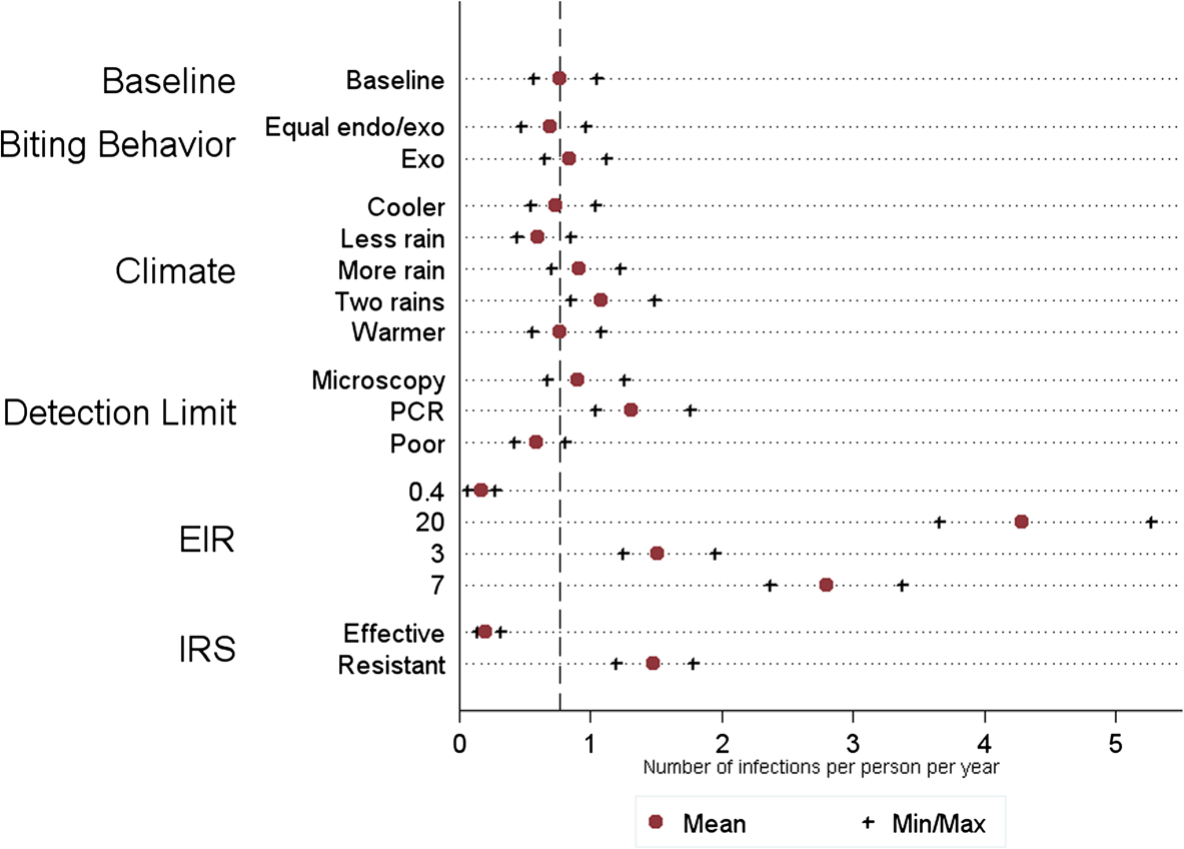
**Summary sensitivity analysis compared to baseline model.** Summary statistics for the effect of changing key parameters on the simulated number of *P. falciparum* infections per person per year averaged across all model variants during the study period of the MTC cohort study (July 2009 – June 2010) as detected by RDT in a population of 10,000 individuals. The simulation ran for two years using 10 seeds for each of the 14 OpenMalaria model variants. The red circles represent the mean and the black plus signs represent the minimum and maximum. The dotted line represents the baseline mean as a measure of comparison.

To examine model sensitivity to changes in the entomological parameters that could occur as a result of different climate patterns an experiment was created to simulate decreased rainfall, increased rainfall, decreased temperature, increased temperature, and two long rainy periods instead of the long and short rains the study area currently experiences. Compared to the baseline, simulating increased rainfall in the same seasonal pattern did not have as great an effect on number of patent infections as did the scenario which increased the short rainy season to match the longer rains. Simulating temperature changes by altering the extrinsic incubation period and resting duration did not have a visible impact on the predicted number of patent infections (Figure 9).

Figure 9 demonstrates the overall results of the one-way sensitivity analysis in relation to the baseline scenario for Rachuonyo South.

## Discussion

### Validation and model analysis

The scenario parameterized for Rachuonyo South district is able to replicate the overall level of prevalence in the given population for the majority of the months out of the year. However, the timing of the peak transmission month is delayed and the depth of the trough in November of the first year of simulations is not captured. Thus, the number of runs simulating the number of patent infections falling within the 95% confidence intervals (CIs) of the observed number of patent infections is lower than optimal. This could be due to several factors, such as inter-annual variation in transmission in the study area.

A main challenge for transmission models is calibration and validation with data from the field. The OpenMalaria transmission model is calibrated primarily by the intensity of malaria transmission, or EIR, for each vector. There are several methods to measure EIR in the field, and the method used varies by location depending on the implementer of the study [37]. Usually the types of surveys necessary to quantify transmission are not done on a regular basis, and in low transmission settings where mosquito densities are low, the longitudinal studies required to estimate EIR are intensive and inherently expensive. Entomological studies with the aim of identifying sporozoite-positive mosquitoes, while important for monitoring vector biting behavior, are not suitable for developing a seasonality pattern for a given total EIR in this area of low, unstable transmission. Perhaps monthly or even weekly studies measuring mosquito density and changes in vector biting behaviour over one or multiple years would be a way of determining the seasonality pattern of transmission.

Despite a clearer picture of overall annual transmission, serology is not able to characterize seasonality of transmission, and without a baseline is unable to give an indication of pre- vs. post- intervention exposure. Serology combined with a seasonality pattern from rainfall data offered a more accurate picture than entomological data alone. While this information can be approximated from weather station data and from remote sensing in areas lacking a weather station, a challenge is relating the amount and seasonality of transmission to the amount of rainfall as their relationship is not linear [38].

The method of evaluation used in this study was to analyse the number of simulation runs which fall within the 95% CIs of the observed data. There is not yet any consensus on how to evaluate uncertainty and goodness-of-fit for model ensembles [39]. The merits of different methods have been discussed for models used in meteorology, climate change and macroeconomics, but questions remain on whether model averaging is appropriate and how to quantify an acceptable level of stochasticity for basing programmatic decisions on model predictions [40]. A consensus should be achieved on these criteria if quantitative projections from such models are to become an integral part of the range of decision-making tools for malaria control.

### Implications of the sensitivity analysis

The sensitivity analysis highlights the robustness of the OpenMalaria transmission model for simulating a range of entomological and epidemiological scenarios. The majority of the simulation results for extreme scenarios of the entomological and biological components of the model remain similar to the simulation results for the baseline scenario, suggesting that small changes in these parameters are unlikely to have a large impact on prevalence, while changes in EIR and effectiveness of IRS have a greater impact on the estimated prevalence in the study area (Figure 9).

### IRS

Pyrethroid resistance has already been documented in western Kenya and elsewhere and much depends on the effectiveness of these insecticides [41-43]. In the study area there are not yet reports of pyrethroid knockdown resistance (kdr) mutations due to the lack of presence of *An. gambiae s.s.,* but there may be other resistance mechanisms present, for example metabolic resistance, given the high numbers of *An. funestus* in the study area. The results of the sensitivity analysis suggest that malaria incidence and prevalence are likely to increase as this resistance continues to rise. As noted in the background section, the Kenya DOMC has alternated deployment of different types of pyrethroid insecticides for different years. While this could potentially have the effect of discouraging resistance to any one formulation, until new insecticides are developed the continued use of only pyrethroids has the potential to encourage resistance.

### Biting behaviour

Initial results of entomological surveys (Cooke, *personal communication)* show evidence of a shift in the relative importance of outdoor biting compared to what has been observed in the neighbouring highland district in the past [10,44]. It is unclear whether this is a behavioral change in response to high LLIN and IRS coverage or whether there have been alterations in overall species composition. For Rachuonyo South there are no baseline data to compare this to. Evidence from lowland districts within Nyanza indicate that both composition and biting behaviour of the malaria vectors has changed over the past five years, coinciding with a substantial scaling up of vector control interventions [12]. Entomological surveys conducted in 2009 – 2011(Stevenson, *personal communication*) show that *An. arabiensis* is now seen more frequently inside and outside dwellings than *An. gambiae s.s.,* the previously-documented major vector in Kisii district [10,44]. Preliminary data from the study sites also indicate that *An. funestus* or other species may be playing an ever increasingly important role on malaria transmission in the area [35].

The observed data more closely resemble the scenario with indoor/outdoor biting profile based on 2009 – 2011 data, which supports the hypothesis of a greater proportion of outdoor biting. If this is the case, there is a limit to the effectiveness of the current vector control interventions in Rachuonyo South (IRS and LLINs) at controlling *P. falciparum* because they target the shrinking proportion of the infective bites occurring indoors. While these interventions will still offer an important level of deterrency, interventions that have a killing effect on exophagic mosquitoes may be an appropriate addition to existing indoor interventions [45]. Larviciding, area repellents, and even interventions targeting the human-stage of the parasite could also be taken into consideration to complement existing methods. Implementation of a number of these methods is currently being piloted in Rachuonyo South (Bousema, *personal communication)*.

### Survey detection limit

The outcome simulated in this scenario is number of patent infections as measured by a Paracheck® rapid test kit manufactured by Orchid Biomedical Systems. The 2010 WHO malaria case management guidelines recommend treatment after parasite-based diagnosis [46]. Quality assurance measures for these tests are based on their ability to detect either 100 or 200 parasites per microliter, not because of the limitations of the RDT technology but rather because of limited accuracy and error of expert microscopy, the “gold” standard in malaria diagnosis in the absence of PCR [47,48]. In addition, there is evidence for changes in the accuracy of diagnosis by RDTs in the East African highlands both over time and across age groups [49].

The implication of the sensitivity of the model to a change in survey detection limit is that if RDTs used in surveys perform poorly, whether the result of low quality manufacturing or improper storage conditions or use, according to simulation results up to 50% of infected individuals would be misclassified. When put in a broader public health context, there are a number of scenarios applicable to the study area when decision-making can be affected by detection limit. These range from a health worker deciding to administer an anti-malarial drug following malaria diagnosis in an individual, to country-wide planning in the public sector health system for estimating quantities of antimalarial drugs required for a given year, to deciding the appropriate time to change the vector control strategy if the measure of transmission is based on an estimate of prevalence.

When approaching a situation where transmission is interrupted, attention must be paid to the type of screening strategy (active vs. passive case detection) and screening method used to detect the last remaining parasitaemia in the population. In these cases the higher presence of asymptomatic, sub-patent infections representing the infectious reservoir of parasites in the population indicates the PCR method would be preferable over a less sensitive method. While molecular diagnostic tools such as PCR and loop-mediated isothermal amplification (LAMP) are both able to detect infections at a much lower parasite density than microscopy or RDTs and may be appropriate in study settings, studies show these methods are not currently suitable for routine diagnosis at a community level [50,51]. However, even the most sensitive PCR diagnostic does not detect all infections in a population. If a large proportion of infections occur at a high parasite density the detection limit of the diagnostic would not be as important a consideration. This sensitivity analysis shows that observed prevalence depends on the method used for detection, a point relevant for study design and modelling alike.

### EIR and climatic patterns

The sensitivity analysis results show that an increase in EIR corresponds to an increase in cases of malaria. The OpenMalaria transmission model is dependent on the length of the gonotrophic cycle of the vector, which is in turn affected by environmental changes. The mosquito resting duration and EIP both decrease as the ambient temperature decreases [33,52,53]. If the EIP duration decreases, a vector infected with *P. falciparum* becomes infectious more quickly. A shorter gonotrophic cycle means both increased biting frequency and increased daily mortality of the vector. The highlands of western Kenya have variable seasonal temperature and rainfall changes; for example, in the late 1990s the study area experienced a resurgence of malaria not seen for decades [54]. Simulation results indicate that changes in temperature resulting in a change in EIP or resting duration and changes in the overall volume of rainfall resulting in a slight change in EIR are not likely to effect the impact of IRS deployment or result in a shift in *P. falciparum* prevalence in the population. In addition, there is preliminary evidence that in the study area increased relative humidity is associated with an increased number of anophelines (Cooke, *personal communication)*. However, even taking into account the caveats for the relationship between malaria transmission and rainfall, changing the pattern of transmission to simulate the effect of an extension of the historically short rainy season to match the rainfall profile of the longer rainy season could result in greater amplitude of incidence in the peak months.

### Limitations

#### Data

Many parameters in the model remain from the initial Tanzanian model parameterization [26], for example the parameters for the mosquito feeding cycle (Additional File 2) and treatment-seeking behaviour (Additional File 3), because there are not yet site-specific studies with this focus. While ample entomological data were collected in the study area, there was less available information on treatment-seeking behaviour and its consequences outside the public sector.

Coartem® was given to all MTC cohort study participants to clear any prevalent *P. falciparum* parasitaemia making it possible to measure malaria incidence at each follow-up. The study excluded pregnant women from the cohort due to the limited data on use in pregnancy and contraindication in the 1^st^ trimester pregnancy of artemether-lumefantrine [19], the active ingredients of Coartem®. Infection with *P. falciparum* during pregnancy has been shown to be associated with increased parasitaemia of the mother due to a weakened immune system as well as an increased likelihood of manifestation of clinical disease in addition to adverse affects on the fetus and newborn [55]. Although this is unlikely to have a major effect on transmission in the population as a whole, the patent infections and uncomplicated episodes in the age groups for women of childbearing age could be underestimated.

#### Model

Since the OpenMalaria transmission model was developed to examine the effect of moderate to high transmission, it does not include a mechanism to account for inter-annual variation in EIR as driven by climatic factors. Thus, every year is treated as the same, which is not the case in the study area. In the western Kenyan highlands sharp increases in incidence occur every few years [54] and are likely to be driven by climate variability; the higher than usual transmission in the cohort following heavy rains during the time of the survey provides an example of such a sharp increase. As a result of the validation using one year’s data in this area of substantial year-to-year variation, firm conclusions are unable to be drawn about the longer-term seasonal transmission in the population.

Nyanza province has the highest prevalence of HIV in Kenya at 15.1% of the population [56,57]. HIV infection increases an individual’s susceptibility to malaria infection and severity of clinical outcomes and decreases immunity [58,59]. A limitation of the transmission model is that it does not account for the interaction between malaria and HIV.

The models analysed here do not explicitly take spatial associations into account. Variation in proximity to breeding sites could be a factor driving the difference in epidemic profile of the study area. The parameterizations used in this study do not take into account the rate of imported cases from lowland areas, as there is frequent travel between the highland Kisii and Nyamira districts and the lowland areas of Rachuonyo North, Nyando and Kisumu districts. Heterogeneity in availability to vectors and imported cases should be taken into account in future simulations of the study area.

## Conclusions

Individual-based stochastic simulations of malaria can be used as a tool to assist decision making for malaria control programmes by testing assumptions about the seasonal pattern of transmission, vector diversity and behavior, and intervention effectiveness in district-level settings. Efforts should be made to ensure models aiding in the understanding of site-specific transmission dynamics are more accessible to programme managers. The sensitivity analysis shows that in order to simulate malaria in the Rachuonyo South highlands, attention must be paid to vector biting behaviour, their susceptibility to IRS, and the detection method used for human surveys. These features will have an impact on predicting the impact of interventions in areas with low and/or variable *P. falciparum* transmission. The sensitivity analysis also demonstrates the accuracy of the model and can lend confidence to end users of these results in informing control options. New methods and tools for analysing and evaluating simulation results will enhance the usefulness of simulations for malaria control decision-making. Measuring EIR through mosquito collection may not be the optimal way to define transmission in areas with low, unstable transmission. Further research into the relationship between different measures of malaria is needed to better quantify transmission in low transmission settings.

## Abbreviations

ACT: Artemisinin Combination Therapy; DDT: Dichlorodiphenyltrichloroethane; EIP: Extrinsic Incubation Period; EIR: Entomological Inoculation Rate; IRS: Indoor Residual Spraying; KEMRI/CDC: Kenya Medical Research Institute/Centers for Disease Control and Prevention; LAMP: Loop-Mediated Isothermal Amplification; LLIN: Long-Lasting Insecticide-Treated Net; MDA: Mass Drug Administration; MTC: Malaria Transmission Consortium; PCR: Polymerase Chain Reaction; RDT: Rapid Diagnostic Test; Swiss TPH: Swiss Tropical and Public Health Institute.

## Competing interests

The authors declare that they have no competing interests.

## Authors’ contributions

EMS designed the experiments, performed the literature review for model parameterization, analysed results and drafted the manuscript. JS participated in parameterization of the model and designed, supervised and conducted the MTC field studies. MC provided field implementation and sample analysis for the MTC entomological field studies. CO and EM supervised and coordinated the field collection of samples. GO was responsible for data management. DH programmed the simulation software. CD provided serological analysis for the MTC field studies. TAS and JC conceived of and designed the study. NC participated in designing the experiments, analysing the results, and drafting the manuscript. All authors read and approved the final manuscript.

## Supplementary Material

Additional file 1 **Title: Model parameterization source overview.** Description: Tables containing a detailed description of the various studies in Rachuonyo South district conducted by MTC and how the data was used to parameterize the base simulation scenario. Click here for file

Additional file 2 **Title: Parameter values for the model of the mosquito feeding cycle.** Description: Tables containing a detailed description of the parameter values and their source(s) for the model of the mosquito feeding cycle. Click here for file

Additional file 3 **Title: Health system parameter values.** Description: Tables containing a detailed description of the parameter values and their source(s) for the model of the health system. Click here for file

Additional file 4 **Title: Description of model demographic parameters.** Description: Tables containing a detailed description of the parameter values and their source(s) for the model of the demography. Click here for file

Additional file 5 **Title: Vector control intervention effective length of protection parameter values.** Description: Tables containing a detailed description of the parameter values and their source(s) for effective length of protection for the model of vector control interventions. Click here for file

Additional file 6 **Title: Vector control intervention effectiveness parameter values.** Description: Tables containing a detailed description of the parameter values and their source(s) for effectiveness for the model of vector control interventions. Click here for file

Additional file 7 **Title: Vector control intervention implementation parameter values.** Description: Tables containing a detailed description of the parameter values and their source(s) for implementation schedule and coverage levels for the model of vector control interventions. Click here for file
