# Supplementary material for: Simulation of malaria epidemiology and control in the highlands of western Kenya
Source: Malar J. 2012 Oct 29;11:357. doi: 10.1186/1475-2875-11-357 (PMC3552835; doi:10.1186/1475-2875-11-357)
Supplement: Additional file 1 — Title: Model parameterization source overview. Description: Tables containing a detailed description of the various studies in Rachuonyo South district conducted by MTC and how the data was used to parameterize the base simulation scenario. [file 1475-2875-11-357-S1.pdf]

Additional File 7: Model parameterization source overview

**Table S7: Model parameterization source overview**	
Total inputs	123
Site-specific MTC data	12 (10%)
Regional/national lit review	41 (33%)
Previous model parameterization	70 (57%)
