# Supplementary material for: Simulation of malaria epidemiology and control in the highlands of western Kenya
Source: Malar J. 2012 Oct 29;11:357. doi: 10.1186/1475-2875-11-357 (PMC3552835; doi:10.1186/1475-2875-11-357)
Supplement: Additional file 2 — Title: Parameter values for the model of the mosquito feeding cycle. Description: Tables containing a detailed description of the parameter values and their source(s) for the model of the mosquito feeding cycle. [file 1475-2875-11-357-S2.pdf]

Additional File 1: Parameter values for the model of the mosquito feeding cycle

Table S1: Parameter values for the model of the mosquito feeding cycle

Parameter	Value
Initial proportion of vectors infected	0.078
Initial proportion of vectors infectious	0.015[1]
Extrinsic incubation period	14**[2-4]
Human blood index	0.97[5]
Proportion of mosquitoes host-seeking on the same day as oviposting	0.313
Probability that the mosquito successfully bites chosen host	0.95
Probability that the mosquito escapes host and finds a resting place after biting	0.95
Probability of a mosquito successfully laying eggs given that it has rested	0.88
Probability of mosquito successfully resting after finding a resting site	0.99
Duration of the resting period of the vector	3[4, 6]
Maximum proportion of day spent host-seeking by vector	0.33
Probability that the mosquito survives the feeding cycle	0.623
Probability that the mosquito successfully bites chosen non-human host	0.95
Probability that the mosquito escapes non-human host and finds a resting place after biting	0.95
Probability of mosquito successfully resting after finding a resting site	0.99
Proportion of encounters on un-protected animals vs. protected animals	1

**Note: detailed description of parameters used in the entomological model can be found in Chitnis 2008[7] and Chitnis 2012[4]. All values based on Chitnis 2012[4] unless otherwise noted.*

References

1. Ndenga B, Githeko A, Omukunda E, Munyekenye G, Atieli H, Wamai P, Mbogo C, Minakawa N, Zhou G, Yan G: **Population dynamics of malaria vectors in western Kenya highlands.** *J Med Entomol* 2006, **43:**200-206.

2. Eling W: **Tropical temperatures can inhibit development of the human malaria parasite Plasmodium falciparum in the mosquito** *Proc Exper Appl Entomol, NEV Amsterdam* 2001, **12:**151 - 156.

3. Noden BH, Kent MD, Beier JC: **The impact of variations in temperature on early Plasmodium falciparum development in Anopheles stephensi.** *Parasitology* 1995, **111 (Pt 5):**539-545.

4. Chitnis N, Hardy D, Smith T: **A Periodically-Forced Mathematical Model for the Seasonal Dynamics of Malaria in Mosquitoes.** *Bulletin of mathematical biology* 2012, **74(5):**1098–1124.

5. Githeko AK, Service MW, Mbogo CM, Atieli FK, Juma FO: **Origin of blood meals in indoor and outdoor resting malaria vectors in western Kenya.** *Acta tropica* 1994, **58:**307-316.

6. Armstrong JA, Bransby-Williams WR: **The maintenance of a colony of Anopheles gambiae, with observations on the effects of changes in temperature.** *Bull World Health Organ* 1961, **24:**427-435.

7. Chitnis N, Smith T, Steketee R: **A mathematical model for the dynamics of malaria in mosquitoes feeding on a heterogeneous host population.** *Journal of Biological Dynamics* 2008, **2:**259 - 285.
