# Supplementary material for: Simulation of malaria epidemiology and control in the highlands of western Kenya
Source: Malar J. 2012 Oct 29;11:357. doi: 10.1186/1475-2875-11-357 (PMC3552835; doi:10.1186/1475-2875-11-357)
Supplement: Additional file 3 — Title: Health system parameter values. Description: Tables containing a detailed description of the parameter values and their source(s) for the model of the health system. [file 1475-2875-11-357-S3.pdf]

Additional File 2: Health system parameter values

Table S2: Health system parameter values

Parameter	Value	Source
National guidelines for first line antimalarial	Coartem	DOMC 2006[1]
National guidelines for inpatient antimalarial	Quinine	DOMC 2006[1]
National guidelines for second line antimalarial	Quinine	DOMC 2006[1]
Initial cure rate in the absence of resistance, first line (%)	0.96	Juma 2008[2]
Initial cure rate in the absence of resistance, inpatient (%)	0.998	Ross 2008[3]
Initial cure rate in the absence of resistance, second line (%)	0.998	Ross 2008[3]
Initial cure rate in the absence of resistance, self-treatment (%)	0.63	Ross 2008[3]
Population complying to drug regimine, ACT (%)	0.892	Kabanywanyi 2010[4]
Population complying to drug regimine, self-treatment (%)	0.85	Ross 2008[3]
Effectiveness of treatment of non-compliers, first line (%)	0.8544	Fogg 2004[3]
Effectiveness of treatment of non-compliers, self-treatment (%)	0	Ross 2008[3]
Probability that a patient with newly incident uncomplicated disease seeks official care per timestep	0.04	Ross 2008[3]
Probability that a patient with uncomplicated disease self-treats per timestep	0.0212	Sumba 2008[5]
Probability that a patient with recurrence of uncomplicated disease seeks official care per timestep	0.04	Ross 2008[3]
Probability that a patient with severe disease obtains appropriate care per timestep	0.48	Ross 2008[3]
Probalility of sequelae in inpatients per timestep, for individuals under 5 years old	0.0132	Ross 2008[3]
Probalility of sequelae in inpatients per timestep, for individuals over 5 years old	0.005	Ross 2008[3]

References

1. DOMC: **National Guidelines for Diagnosis, Treatment and Prevention of Malaria in Kenya.** (Management DoMCC ed.; 2006.

2. Juma EA, Obonyo CO, Akhwale WS, Ogutu BR: **A randomized, open-label, comparative efficacy trial of artemether-lumefantrine suspension versus artemether-lumefantrine tablets for treatment of uncomplicated Plasmodium falciparum malaria in children in western Kenya.** *Malar J* 2008, **7:**262.

3. Ross A, Penny M, Maire N, Studer A, Carneiro I, Schellenberg D, Greenwood B, Tanner M, Smith T: **Modelling the epidemiological impact of intermittent preventive treatment against malaria in infants.** *PLoS One* 2008, **3:**e2661.

4. Kabanywanyi AM, Lengeler C, Kasim P, King'eng'ena S, Schlienger R, Mulure N, Genton B: **Adherence to and acceptability of artemether-lumefantrine as first-line anti-malarial treatment: evidence from a rural community in Tanzania.** *Malar J* 2010, **9:**48.

5. Sumba PO, Wong SL, Kanzaria HK, Johnson KA, John CC: **Malaria treatment-seeking behaviour and recovery from malaria in a highland area of Kenya.** *Malar J* 2008, **7:**245.
