# Supplementary material for: Simulation of malaria epidemiology and control in the highlands of western Kenya
Source: Malar J. 2012 Oct 29;11:357. doi: 10.1186/1475-2875-11-357 (PMC3552835; doi:10.1186/1475-2875-11-357)
Supplement: Additional file 4 — Title: Description of model demographic parameters. Description: Tables containing a detailed description of the parameter values and their source(s) for the model of the demography. [file 1475-2875-11-357-S4.pdf]

Additional File 3: Description of model demographic parameters

Table S3: Description of model demographic parameters and source

**Age Group**	**<1**	**1-4**	**5-9**	**10-14**	**15-19**	**20-24**	**25-29**	**30-34**	**35-39**	**40-44**	**45-49**	**50-54**	**55-59**	**60-64**	**65-69**	**70-74**	**75-79**	**80-84**	**85+**
**Distribution (%)[1]**	2.6	13.1	15.2	13.8	10	8.6	702	6	4.7	4.1	3.5	2.9	2.2	1.9	1.3	1.1	0.6	1	0.1
**Distribution (#)***	510	2,567	2,979	2,705	1,960	1,685	1,411	1,176	921	804	686	568	431	372	255	216	118	196	20

**Case Fatality Rate****	<3 months: 0.09189<br>3-8 months: 0.0810811<br>9-17 months: 0.0648649	18-30 months: 0.0689189<br>2.5-3.5 years: 0.0675676<br>3.5-4.5 years: 0.0297297	4.5-7.5 years: 0.0459459<br>7.5-12.5 years: 0.0945946<br>12.5-14 years: 0.1243243

*Proportion at national level applied to census total population of study area, 19,598 individuals
**Deaths among hospitalized cases of severe malaria. Schellenberg, 1999[2]

References

1. Kenya National Bureau of Statistics (KNBS) and ICF Macro. 2010. **Kenya Demographic and Health Survey 2008-09.** Calverton, Maryland: KNBS and ICF Macro.

2. Schellenberg D, Menendez C, Kahigwa E, Font F, Galindo C, Acosta C, Armstrong Schellenberg J, Aponte JJ, Kimario J, Urassa H, Mshinda H, Tanner M, Alonso P: **African children with malaria in an area of intense *Plasmodium falciparum* transmission: features on admission to the hospital and risk factors for death.** *Am J Trop Med Hyg* 1999, **63:**431 – 438.
