# Supplementary material for: Simulation of malaria epidemiology and control in the highlands of western Kenya
Source: Malar J. 2012 Oct 29;11:357. doi: 10.1186/1475-2875-11-357 (PMC3552835; doi:10.1186/1475-2875-11-357)
Supplement: Additional file 5 — Title: Vector control intervention effective length of protection parameter values. Description: Tables containing a detailed description of the parameter values and their source(s) for effective length of protection for the model of vector control interventions. [file 1475-2875-11-357-S5.pdf]

Additional File 4: Vector control intervention effective length of protection parameter values

Table S4: Vector control intervention effective length of protection parameter values*

Parameter	Mean	Sigma	L	Function	k
ITN Hole Rate	0	0.8	-	-	-
ITN Rip Rate	2.7	0.8	-	-	-
ITN Initial Insecticide	1	0	-	-	-
ITN Insecticide Decay	0	0	3[1]	exponential[2]	-
ITN Attrition	-	-	15.57941	constant	18
IRS Decay	-	-	0.33	exponential[2]	-

*Note: all values are based on Chitnis 2010[13] updated with the model described in Briët 2012[3] unless otherwise noted.

References

1. Chitnis N, Smith T, Schapira A: **Parameter Values for Transmission Model. Unpublished work.** pp. 1 - 17. Basel: Swiss TPH; 2010:1 - 17.

2. Ombok MO, G; Bayoh, N; Vulule, J; Gimnig, J; Walker, E: **Entomological monitoring of the indoor residual spraying (IRS) program in western Kenya.** In *Kenya National Malaria Forum* Nairobi, Kenya; 2011.

3. Briet OJ, Hardy D, Smith TA: **Importance of factors determining the effective lifetime of a mass, long-lasting, insecticidal net distribution: a sensitivity analysis.** *Malaria journal* 2012, **11:**20.
