# Supplementary material for: Simulation of malaria epidemiology and control in the highlands of western Kenya
Source: Malar J. 2012 Oct 29;11:357. doi: 10.1186/1475-2875-11-357 (PMC3552835; doi:10.1186/1475-2875-11-357)
Supplement: Additional file 6 — Title: Vector control intervention effectiveness parameter values. Description: Tables containing a detailed description of the parameter values and their source(s) for effectiveness for the model of vector control interventions. [file 1475-2875-11-357-S6.pdf]

**Additional File 5:** Vector control intervention effectiveness parameter values

**Table S5: Vector control intervention effectiveness parameter values***

| **Parameter** | **Deterrency** | **Preprandial Killing Effect** | **Postprandial Killing Effect** |
|---|---|---|---|
| ITN Base Factor | - | 0 | 0 |
| ITN Hole Factor | 1 | 0 | 0 |
| ITN Hole Scaling Factor | 1 | 0 | 0 |
| ITN Insecticide Factor | 8416 | 972 | 972 |
| ITN Insecticide Scaling Factor | 0.001 | 0.001 | 0.001 |
| ITN Interaction Factor | 1 | 0 | 0 |
| IRS Pyrethroid Insecticide | 0.1116 | 0 | 0.2772 |

**Note: all values are based on Chitnis 2010[1] updated with the model described in Briet 2012[2] unless otherwise noted. Please note that all figures for "IRS Pyrethroid Insecticide" in this table have been adjusted from their original levels to account for the proportion of exposure in the study area occurring outdoors. Unadjusted initial values for these parameters by species can be found in the sources noted above.*

## References


1. Chitnis N, Smith T, Schapira A: **Parameter Values for Transmission Model. Unpublished work.** pp. 1 - 17. Basel: Swiss TPH; 2010:1 - 17.

2. Briet OJ, Hardy D, Smith TA: **Importance of factors determining the effective lifetime of a mass, long-lasting, insecticidal net distribution: a sensitivity analysis.** *Malaria journal* 2012, **11:**20.
