# Supplementary material for: Simulation of malaria epidemiology and control in the highlands of western Kenya
Source: Malar J. 2012 Oct 29;11:357. doi: 10.1186/1475-2875-11-357 (PMC3552835; doi:10.1186/1475-2875-11-357)
Supplement: Additional file 7 — Title: Vector control intervention implementation parameter values. Description: Tables containing a detailed description of the parameter values and their source(s) for implementation schedule and coverage levels for the model of vector control interventions. [file 1475-2875-11-357-S7.pdf]

Additional File 6: Vector control intervention implementation parameter values

Table S6: Vector control intervention implementation parameter values*

Intervention	Usage	Target age (years)	Timing	Coverage
ANC	1	0.0833[1]	0	0.8[1]
Mass	-	-	0	0.3026
			2009 April	
IRS	-	-	2010 June	0.693
MDA	-	cohort	2009 June	0.15

**Note: all values are based on MTC cohort data unless otherwise noted.*

References

1. Chitnis N, Smith T, Schapira A: **Parameter Values for Transmission Model. Unpublished work.** pp. 1 - 17. Basel: Swiss TPH; 2010:1 - 17.
